# Effect of Crude Oil on Growth, Oxidative Stress and Response of Antioxidative System of Two Rye (*Secale cereale* L.) Varieties

**DOI:** 10.3390/plants10010157

**Published:** 2021-01-14

**Authors:** Liubov Skrypnik, Pavel Maslennikov, Anastasia Novikova, Mikhail Kozhikin

**Affiliations:** Institute of Living Systems, Immanuel Kant Baltic Federal University, Universitetskaya str. 2, 236040 Kaliningrad, Russia; PMaslennikov@kantiana.ru (P.M.); AENovikova@stud.kantiana.ru (A.N.); MKozhikin1@kantiana.ru (M.K.)

**Keywords:** petroleum, environmental pollution, lipid peroxidation, chlorophyll *a*, chlorophyll *b*, secondary metabolites, Foyer–Halliwell–Assad cycle

## Abstract

Rye (*Secale cereale* L.) is one of the most important cereal crops in Eastern and Northern Europe, showing better tolerance to environmental stress factors compared to wheat and triticale. Plant response to the crude oil-polluted soil depends on plant species, oil concentration, time of exposure, etc. The current study is aimed at investigating the growth, oxidative stress and the response of antioxidative system of two rye varieties (Krona and Valdai) cultivated on crude oil-contaminated soils at different concentrations (1.5, 3.0, 6.0, and 12.0%). Inhibition of rye growth was observed at crude oil concentrations of above 3% for above-ground plant parts and of above 1.5% for roots. A decrease in content of chlorophyll *a* and total chlorophylls in Krona variety was detected at 1.5% oil concentration in soil and in Valdai variety at 3% oil concentration. Compared with the control, the content of malondialdehyde was significantly increased in the Krona variety at 3% oil concentration and in Valdai variety at 6% oil concentration. The crude oil-induced oxidative stress was minimized in rye plants by the enhanced contents of low-molecular antioxidants (proline, non-protein thiols, ascorbic acid, phenolic compounds) and activities of superoxide dismutase, catalase, ascorbate peroxidase, and glutathione peroxidase. The strongest positive correlation was detected between the content of malondialdehyde and contents of proline (r = 0.89–0.95, *p* ≤ 0.05) and phenolic compounds (r = 0.90–0.94, *p* ≤ 0.05) as well as superoxide dismutase activity (r = 0.81–0.90, *p* ≤ 0.05). Based on the results of a comprehensive analysis of growth and biochemical parameters and of the cluster analysis, Valdai variety proved to be more resistant to oil pollution. Due to this, Valdai variety is considered to be a promising rye variety for cultivation on moderately oil-polluted soils in order to decontaminate them. At the same time, it is necessary to conduct further studies aimed at investigating oil transformation processes in the soil-rye system, which would make it possible to determine the efficiency of using this cereal for soil remediation.

## 1. Introduction

Crude oil (petroleum) hydrocarbons are one of the most common groups of persistent organic pollutants [1,2,3]. Petroleum hydrocarbons are known to be toxic to many living organisms due to their mutagenic and carcinogenic properties [4,5]. The low rate of decomposition of oil and oil products in the environment triggers their accumulation and a gradual increase in their concentration in the environmental objects, including the soil. After getting into the soil, crude oil products destroy its structure, upset the air–water balance [1], alter the soil physicochemical properties [6,7], inhibit the microbial proliferation [1], disrupt the soil enzymatic activity [8,9], and have a negative impact on terrestrial and soil mesofauna [6], as well as on plant growth and development [1,2,3,4,5].

The growth and development disorder of plants, growing on oil-contaminated soils, is caused by several reasons. Firstly, the absorption of toxic petroleum molecules by plants can modify the permeability and structure of the plasma membrane [6], alter the shape and size of the parenchyma tissue, reduce the intercellular space in the cortex of the stem and roots, and inhibit the mitotic activity of the root meristem [10]. Secondly, insufficient aeration caused by air displacement from the pore spaces between the soil particles by crude oil leads to root stress and low water availability to the plant [11]. Moreover, oil pollution minimizes the percentage of organic matter available to plants and reduces the amount of mineral nutrients such as sodium, phosphates, potassium, sulfates, and nitrates [8,9].

The response of plants to oil pollution can manifest itself at various levels (physiological, biochemical, and molecular). Detailed information about the effect of oil contamination on photosynthetic activity is scarce. For example, it has recently been reported that crude oil pollution reduces overall photosynthetic activity and chlorophyll contents in plants [9,11]. Notable symptoms observed in plants growing on oil-polluted soil also include a decrease in the activity of starch metabolizing enzymes [9] and a decrease in content of total carbohydrates, total proteins, and total amino acids [9,12]. The most dangerous disorder, resulted from the effect of pollutants, including crude oil and petroleum hydrocarbons, on plants, is oxidative stress, which leads to the formation of many reactive oxygen species (ROSs) with high oxidizing capacity in cells (superoxide radical (O_2_^•−^), H_2_O_2_, hydroxyl radical (^•^OH), etc.). In one respect, the ROSs destroy cell-membrane complexes, disrupt transport processes and intracellular reactions, and thereby inhibit growth activity [13,14,15]. Contrastingly, plants use ROS as a second messenger in many signal transduction cascades, and therefore ROS accumulation is essential to plant development and defense [16]. For these reasons, the plant antioxidative defense network plays a crucial role in controlling the lifetime of the ROS signals and preventing uncontrolled oxidation.

Despite the fact that the antioxidative system and its significance for the adaptation of plants to pollution stresses has been reviewed frequently, little is known about the effects of crude oil stress on the plant antioxidative system. Some studies have shown a change in the contents of proline, non-protein thiols [5,17], ascorbic acid, riboflavin, anthocyanins [18], phenolic compounds, and flavonoids [19] in plants growing on oil contaminated soils. A number of authors have investigated the effect of oil pollution on the activity of plant antioxidative enzymes [9,12]. Taking into consideration the fact that the antioxidative status of plants may directly affect their adaptation to environmental stress, it is necessary to make a detailed study of crude oil’s effects on the plant antioxidative system.

Rye (*Secale cereale* L.) is one of the most important cereal crops in Eastern and Northern Europe. Recently, interest in rye production has increased because of its nutritional value and its tolerance to environmental stress factors. Rye proves to be a rich source of phytochemicals such as phenolic acids, flavonoids, anthocyanins, lignans, alkylresorcinols, and benzoxazinoids [20,21]. Unlike wheat and triticale, rye is more tolerant to abiotic and biotic stress factors, cold resistant, and able to grow on nutrient-poor sandy soils with a low pH [22].

Most of the previous studies, which were devoted to the investigation of the oil pollution effect on plant growth and resistance, examined the plants growing in coastal and oilfield areas, and determined their phytoremediation potential [23,24,25,26]. There is comparatively little information available on the effect of oil pollution on crop plants [5,11,27,28,29,30]. To the best of our knowledge, the current study will be one of the earliest studies aimed at investigating the effects of different concentrations of crude oil in the soil on growth, oxidative stress, and antioxidative response in rye plants. Moreover, since genotypes within the species are known to vary in their resistance to environmental stress, two rye varieties were chosen for the current study due to their resistance to low temperatures, soil acidity, and increased concentration of aluminum ions [31,32,33]. The results of the current study will contribute to an increased knowledge about (i) the growth and physiological responses of two rye varieties to various levels of oil pollution in the soil, (ii) oxidative stress induced in rye plants in conditions of soil pollution with crude oil, and (iii) the reaction of low molecular weight antioxidants and antioxidative enzymes in two rye varieties growing on the oil-contaminated soil. The findings of the present study will enhance understanding of the mechanism of oil tolerance in cereals and will make it possible to determine a rye variety that tends to be more resistant to oil pollution.

## 2. Results

### 2.1. Effect of Toxic Concentrations of Crude Oil on Plant Growth and Biomass of Rye Varieties

Soil pollution with crude oil has a significant impact on plant growth processes. The current study revealed that rye response to crude oil-polluted soil depended on the plant variety and oil concentrations in the soil (Table 1).

Krona variety appeared to be more sensitive to oil concentration in the soil. An evidential decrease in shoot fresh weight was observed at 6% oil concentration in the soil, and a decrease in shoot dry weight was detected at 3% oil concentration in the soil.

Valdai variety demonstrated a significant decrease in shoot fresh weight at 12% oil concentration in the soil. In addition, the stimulating effect of low oil concentrations (1.5 and 3.0%) on the biomass of this rye variety was determined. This was most notable when the plant dry weight was analyzed. At the given oil concentrations in the soil, this parameter turned out to be about 1.2 times higher in comparison with the control (without oil introduction). Water content in shoots of both Krona and Valdai rye varieties decreased with an increase in the oil concentration in the soil. However, a significantly lower value of this parameter was recorded only at the maximum oil concentration equal to 12% (Table 1).

The root system of the studied rye varieties proved to be even more affected by crude oil contamination. Unlike above-ground plant parts, inhibition of root growth was observed at lower oil concentrations in the soil. This tendency was characteristic of both the Krona and Valdai rye varieties. A significant decrease in both root fresh weight and dry weight compared to the control was recorded at 1.5% oil concentration in the Krona variety. The Valdai variety demonstrated a decrease in root fresh weight at 1.5% oil concentration in the soil and a decrease in root dry weight at 3.0% oil concentration in the soil. An evidential decrease in water content in roots of both rye varieties was recorded only at the maximum oil concentration in the soil equal to 12%.

Generally, at the maximum oil concentration in the soil equal to 12%, the above-ground shoot fresh weight was 2.3 times lower in the Krona variety and, respectively, 1.3 times lower in the Valdai variety compared to the control. At the same time, the root dry weight was 4.1 times (Krona variety) and 3.3 times (Valdai variety) lower than that of the control plants.

### 2.2. Effect of Toxic Concentrations of Crude Oil on Chlorophylls and Carotenoids of Rye Varieties

**Figure 1:**
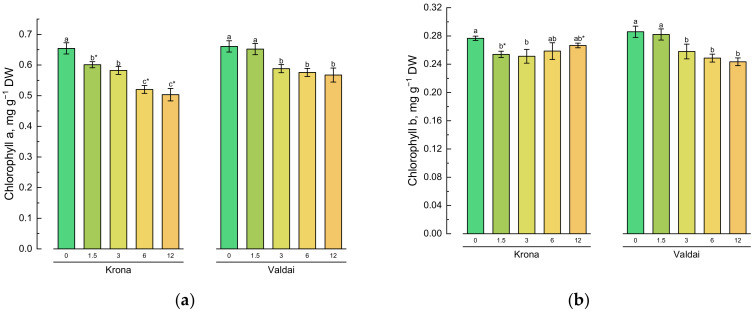

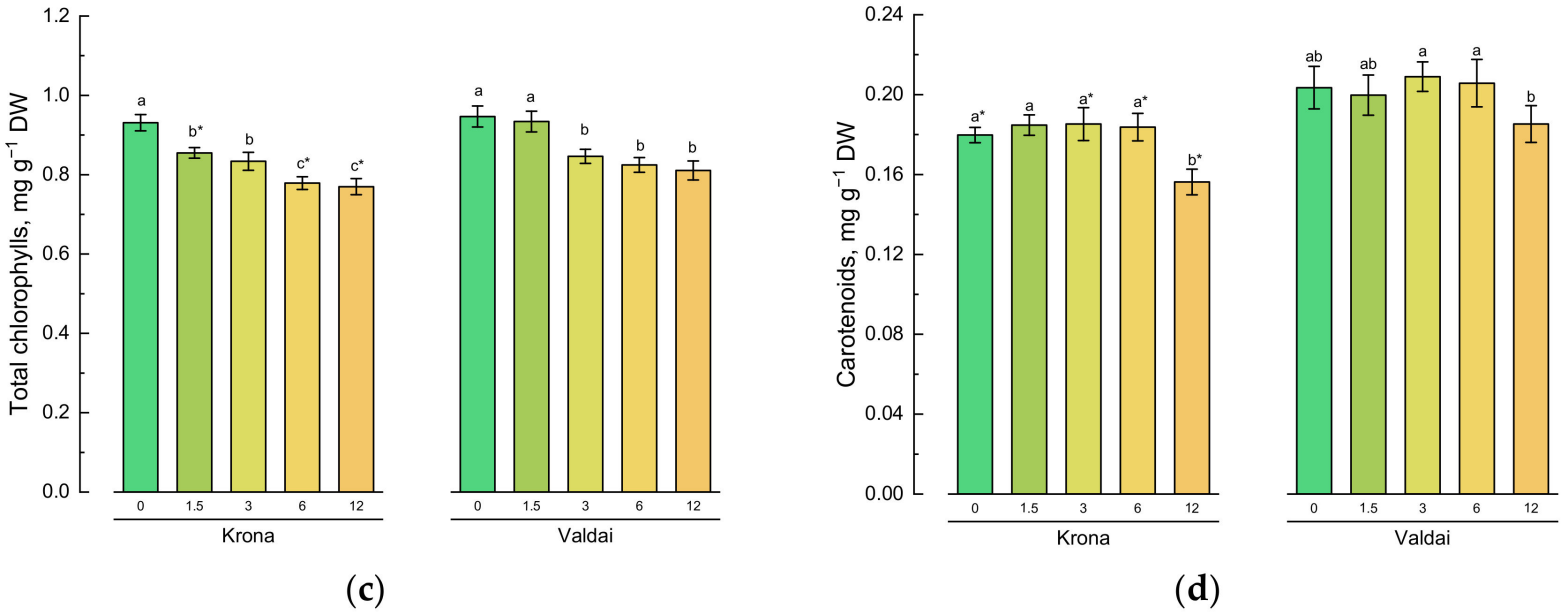
Effect of soil pollution with crude oil (given on *X*-axis in %) on contents of photosynthetic pigments: (**a**) chlorophyll *a*, (**b**) chlorophyll *b*, (**c**) total chlorophylls, and (**d**) carotenoids in shoots of two rye varieties. Different lower-case letters indicate significant differences between plants by oil concentration in the soil for each variety separately, and asterisks * indicate significant differences between rye varieties at *p* ≤ 0.05 based on post hoc Tukey’s test (*n* = 4).

Oil contamination of the soil led to significant decrease in contents of chlorophyll *a*, chlorophyll *b* and total chlorophylls in Krona shoots even at low concentrations (1.5%). The Valdai variety demonstrated significant changes at oil concentrations equal to 3% or more (Figure 1a–c). The decrease in contents of chlorophyll *a* and total chlorophylls in conditions of soil pollution with crude oil was more significant in Krona plants than in Valdai plants (Figure 1a,c).

An opposite result was observed for content of chlorophyll *b* in rye shoots. A lower pigment content was recorded in Krona plants grown at 1.5 and 3% oil concentrations in the soil compared to the control. However, at higher levels of oil concentration, there were no significant differences compared to the control. On the contrary, the Valdai variety demonstrated a decrease in the content of chlorophyll *b* at an oil concentration in the soil equal to 3% or more (Figure 1b).

The content of carotenoids in Krona shoots decreased significantly only at 12% oil concentration in the soil (Figure 1c). Even at the maximum oil concentration in the soil equal to 12%, no significant changes in the content of carotenoids in the Valdai variety were detected in comparison with the control.

### 2.3. Effect of Toxic Concentrations of Crude Oil on Oxidative Stress Parameters in Rye Varieties

**Figure 2:**
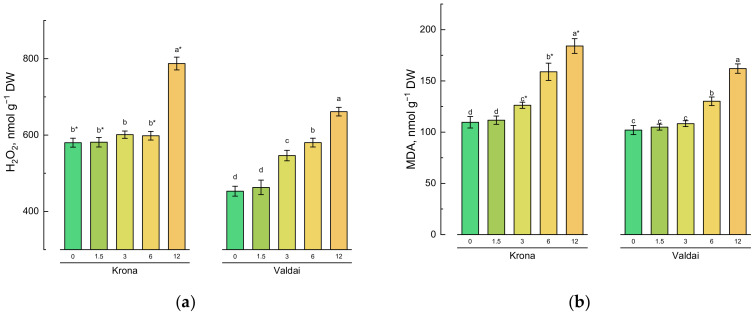
Effect of soil pollution with crude oil (given on *X*-axis in %) on (**a**) hydrogen peroxide content and (**b**) malondialdehyde (MDA) content in shoots of two rye varieties. Different lower-case letters indicate significant differences between plants by oil concentration in the soil for each variety separately, and asterisks * indicate significant differences between rye varieties at *p* ≤ 0.05 based on post hoc Tukey’s test (*n* = 4).

Contents of hydrogen peroxide content and malondialdehyde, which are the products of lipid peroxidation, were used to determine induction of oxidative stress resulted from soil oil pollution. Hydrogen peroxide content in rye shoots depended on the oil concentration in the soil and rye variety (Figure 2a; Appendix A, Table A1). A significantly higher level of H_2_O_2_ was recorded only at the maximum oil concentration (12%) in the Krona variety. Hydrogen peroxide content in Valdai plants was lower than in Krona plants. However, essential changes in its content in comparison with the control were recorded at 3% oil concentration in the soil (Figure 2a).

Malondialdehyde (MDA) accumulation in plants grown on the crude oil-polluted soil also depended on the rye variety (Figure 2b). Thus, for the Krona variety, a significant increase in MDA was recorded in plants grown on the soil whose oil concentration was equal to 3% or more. A significant increase in MDA production was observed in Valdai variety plants grown on the soil whose oil concentration was equal to 6% or more. On average, at the maximum oil concentration in the soil (12%), MDA content in plants was approximately 1.6 times higher than in the control.

### 2.4. Effect of Toxic Concentrations of Crude Oil on Non-Enzymatic Antioxidants of Rye Varieties

**Figure 3:**
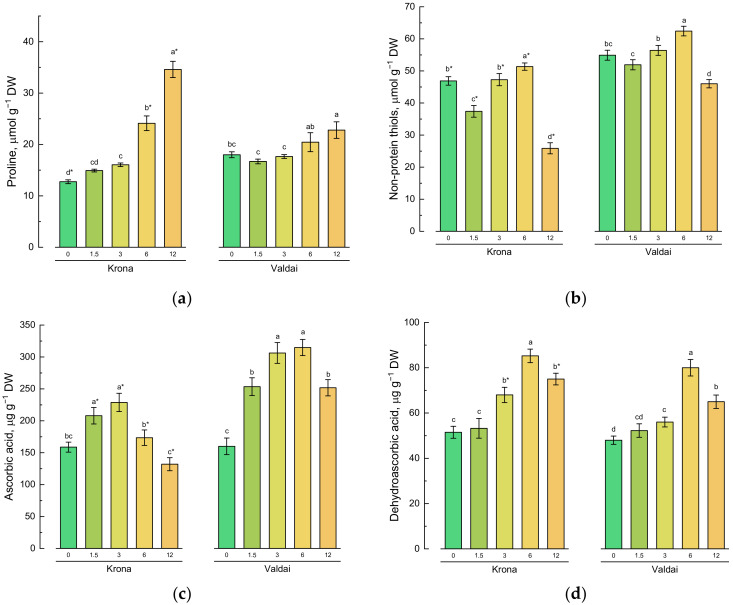

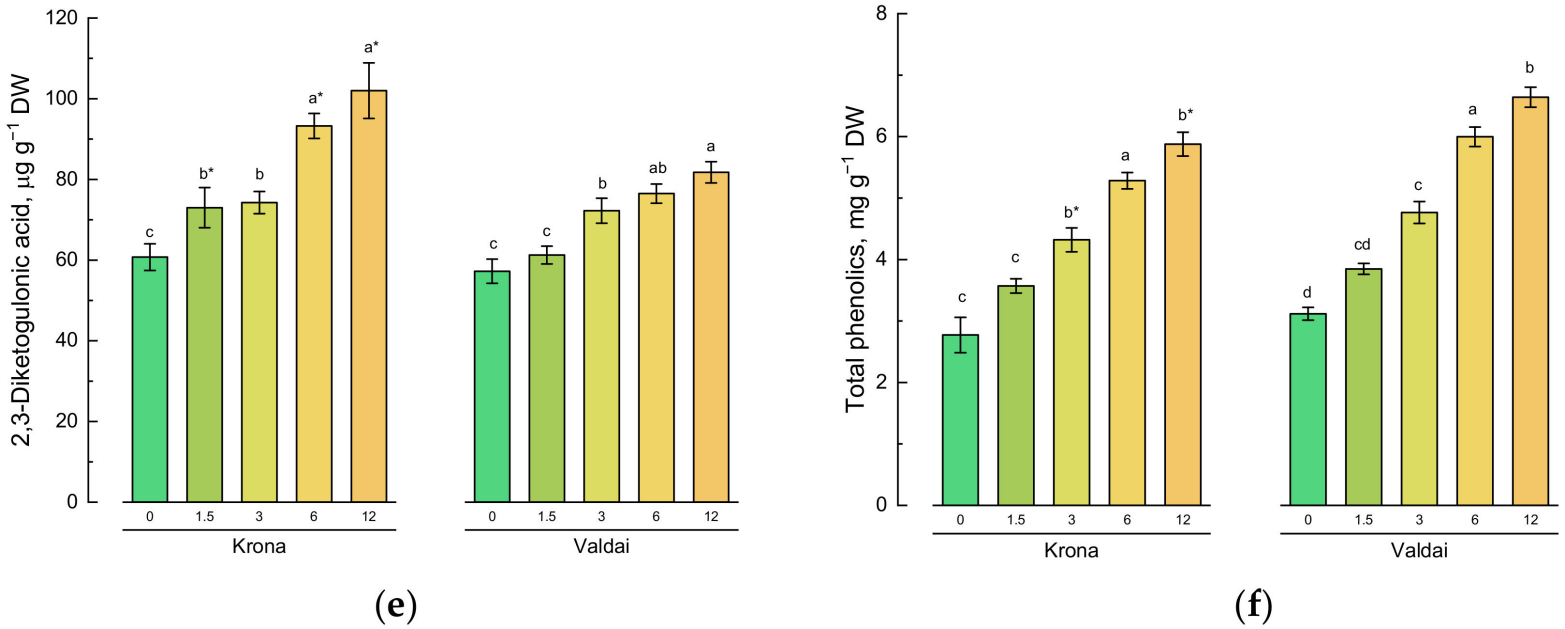
Effect of soil pollution with crude oil (given on *X*-axis in %) on contents of non-enzymatic antioxidants: (**a**) proline, (**b**) non-protein thiols, (**c**) ascorbic acid, (**d**) dehydroascorbic acid, (**e**) 2,3-diketogulonic acid, and (**f**) total phenolic compounds in shoots of two rye varieties. Different lower-case letters indicate significant differences between plants by oil concentration in the soil for each variety separately, and asterisks * indicate significant differences between rye varieties at *p* ≤ 0.05 based on post hoc Tukey’s test (*n* = 4).

Soil pollution with crude oil led to an increase in the content of proline in the shoots of both rye varieties in comparison with the control (Figure 3a). However, more significant changes took place in Krona shoots. Thus, at 12% oil concentration in the soil, proline content in shoots of this variety was approximately 2.6 times higher than in the control. Proline content of Valdai variety was only 1.2 times higher compared to the control.

Changes in the content of non-protein thiols differed from changes in the content of proline in conditions of soil pollution with crude oil (Figure 3b). At low oil concentrations (1.5 and 3%), content of non-protein thiols in the shoots of both varieties decreased in comparison with the control. At 6% oil concentration in the soil, the maximum non-protein thiols content was observed in both varieties while at the maximum oil concentration equal to 12%, non-protein thiols content in shoots was minimal.

A sharp increase in the content of ascorbate was detected at minor oil concentrations in the soil (1.5 and 3%) in Krona shoots, and at all studied oil concentrations in Valdai shoots in comparison with the control (Figure 3c). Contents of dehydroascorbic acid (DHA) and 2,3-diketogulonic acid (DKGA) in rye shoots were about 3 times less than content of ascorbic acid (Figure 3d,e). The highest DHA content for both rye varieties was observed at 6% oil concentration in the soil. DKGA content increased with the increase in oil concentration in the soil and was maximal at the maximum pollution level.

Phenolic compounds accumulation increased by 2.1–2.2 times in shoots of both varieties grown on the soil having 12% oil concentration compared to the control (Figure 3f).

### 2.5. Effect of Toxic Concentrations of Crude Oil on Activities of Antioxidative Enzymes in Rye Varieties

**Figure 4:**
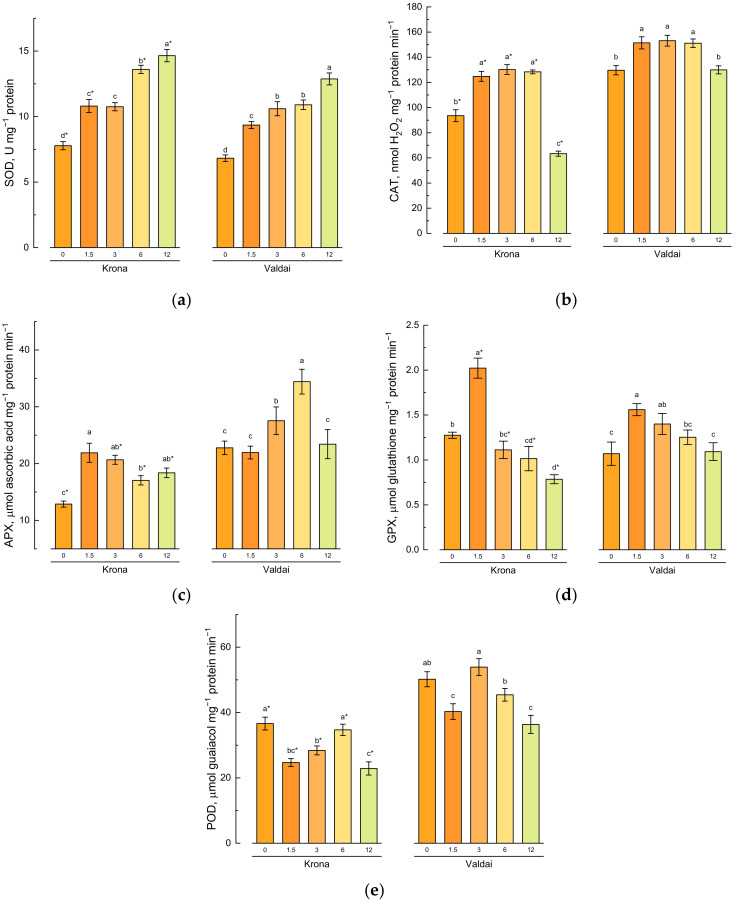
Effect of soil pollution with crude oil (given on *X*-axis in %) on the activities of antioxidative enzymes: (**a**) superoxide dismutase (SOD), (**b**) catalase (CAT), (**c**) ascorbate peroxidase (APX), (**d**) glutathione peroxidase (GPX) and (**e**) peroxidase (POD) in shoots of two rye varieties. Different lower-case letters indicate significant differences between plants by oil concentration in the soil for each variety separately, and asterisks * indicate significant differences between rye varieties at *p* ≤ 0.05 based on post hoc Tukey’s test (*n* = 4).

Soil pollution with crude oil led to an increase in superoxide dismutase activity in shoots of both varieties (Figure 4a). A significant increase in enzyme activity was observed even at 1.5% oil concentration in the soil. The maximum activity was recorded in plants grown on the soil having 12% oil concentration.

Catalase activity in shoots of both varieties also increased at oil concentrations equal to 1.5%, 3%, and 6% in comparison with the control (Figure 4b). However, at the maximum oil concentration (12%), a sharp decrease in catalase activity was observed. In this part of the experiment, catalase activity in Krona shoots was minimal, and it was comparable to the control in Valdai shoots.

Changes in ascorbate peroxidase (APX) activity depending on the oil concentration in the soil differed in shoots of different varieties (Figure 4c). Thus, ascorbate peroxidase activity in Krona plants grown on the oil-polluted soil was significantly higher than that of the control. Compared to the control, a higher ascorbate peroxidase activity was detected in Valdai plants grown only on the soil having 3% and 6% oil concentrations.

The maximum glutathione peroxidase (GPX) activity was detected in shoots of both varieties at 1.5% oil concentration in the soil (Figure 4d). A decrease in the activity of this enzyme was observed at higher oil concentrations. GPX activity in Krona variety was 1.6 times lower than in the control at the maximum oil concentration in the soil equal to 12%. GPX activity in Valdai variety did not differ significantly from the control at the maximum oil concentration in the soil.

Peroxidase (POD) activity in rye shoots depended on the oil concentration in the soil and rye variety (Figure 4e, Appendix A, Table A2). This enzyme activity was lower in the Krona variety than in the control at low oil concentrations in the soil (1.5 and 3%) and at the maximum oil concentration (12%). At 6% oil concentration in the soil, peroxidase activity in shoots of this variety was equitable to the control. A significantly lower peroxidase activity compared to the control was recorded in the Valdai variety at the lowest studied oil concentration in the soil equal to 1.5% and at the highest studied oil concentration equal to 12%. In other variants of the experiment, no significant differences were detected between the control and the plants grown on the crude oil-polluted soil.

### 2.6. Relationship between Growth Parameters, Oxidative Stress and Antioxidative Response of Rye Varieties

**Figure 5:**
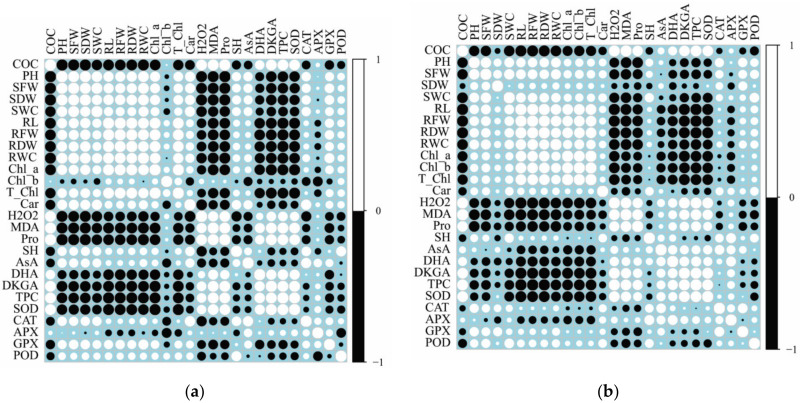
Pearson correlation plots for the studied growth parameters (COC—crude oil concentration in soil, PH—plant height, SFW—shoot fresh weight, SDW—shoot dry weight, SWC—shoot water content, RL—root length, RFW—root fresh weight, RDW—root dried weight, RWC—root water content), biochemical (Chl_a—chlorophyll *a*, Chl_b—chlorophyll *b*, T_Chl—total chlorophylls, Car—carotenoid, MDA—malondialdehyde, H_2_O_2_—hydrogen peroxide, Pro–proline, SH–non-protein thiols, AsA–ascorbic acid, DHA–dehydroascorbic acid, DKGA–2,3-diketogulonic acid, and TPC–total phenolic compounds), and enzymatic parameters (SOD–superoxide dismutase, CAT–catalase, APX–ascorbate peroxidase, GPX–glutathione peroxidase, POD–peroxidase) of Krona variety (**a**) and Valdai variety (**b**).

A Pearson’s correlation analysis was executed between different studied parameters of two rye varieties (Figure 5). In both Krona and Valdai varieties, contents of MDA, H_2_O_2_, proline, dehydroascorbic acid, 2,3-diketogulonic acid, and phenolic compounds, as well as activity of SOD positively correlated with the oil concentration in soil (r > 0.5, *p* < 0.05). The negative correlation was detected between contents of MDA and H_2_O_2_ and growth-related parameters, contents of chlorophyll *a*, total chlorophylls, carotenoids. Additionally, in the Krona variety, contents of non-protein thiols, ascorbic acid and activities of CAT and GPX were negatively correlated with parameters of oxidative stress (r = −0.51 to −0.95, *p* < 0.05).

The relationship between content of non-enzymatic antioxidants and activities of antioxidative enzymes differed in shoots of both rye varieties (Figure 5a,b). In the Krona variety, strong positive correlation (r > 0.75, *p* < 0.05) was detected between SOD activity and contents of proline, dehydroascorbic acid, 2,3-diketogulonic acid, and phenolic compounds; POD activity and content of non-protein thiols; APX activity and content of ascorbic acid. In the Valdai variety, strong positive correlation (r > 0.75, *p* < 0.05) was detected between SOD activity and contents of dehydroascorbic acid and phenolic compounds; APX activity and contents of non-protein thiols and dehydroascorbic acid.

### 2.7. Heat Map and Cluster Analysis of Growth Parameters, Oxidative Stress and Antioxidative Response of Rye Varieties at Different Concentration of Crude Oil in Soil

**Figure 6:**
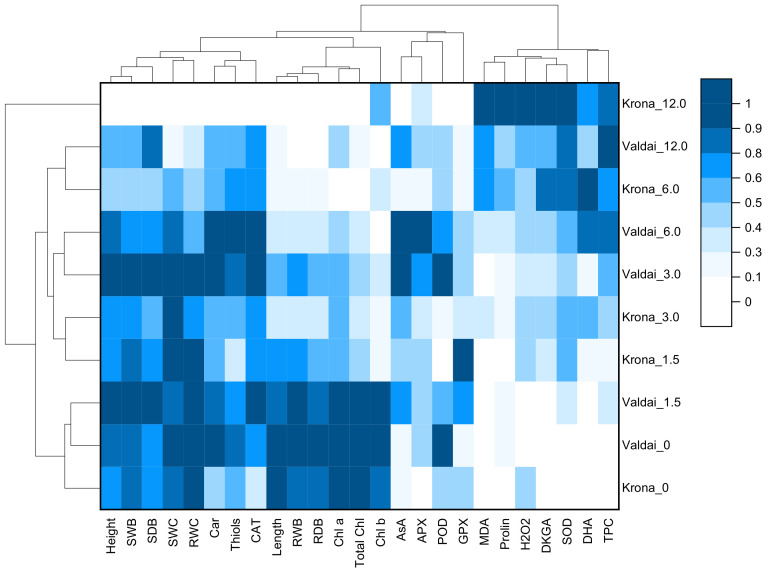
Heat map with clusters for studied parameters (at the top) and rye varieties grown on soils with different concentration of crude oil (at the left). PH—plant height, SFW—shoot fresh weight, SDW—shoot dry weight, SWC—shoot water content, RL—root length, RFW—root fresh weight, RDW—root dried weight, RWC—root water content, MDA—malondialdehyde, H_2_O_2_—hydrogen peroxide, Chl_a—chlorophyll *a*, Chl_b—chlorophyll *b*, T_Chl—total chlorophylls, Car—carotenoid, Pro–proline, SH–non-protein thiols, AsA–ascorbic acid, DHA–dehydroascorbic acid, DKGA–2,3-diketogulonic acid, and TPC–total phenolic compounds, SOD-superoxide dismutase, CAT–catalase, APX–ascorbate peroxidase, GPX–glutathione peroxidase, POD–peroxidase.

Based on the normalized values of studied parameters, a heat map with cluster analysis was performed (Figure 6). The dendrogram presented in Figure 6 (top) demonstrates that all the studied parameters can be divided into four clusters.

The first cluster included such parameters as plant height, shoot fresh weight, shoot dry weight, shoot water content, root water content, carotenoid content, non-protein thiols content, and catalase activity. In this cluster, parameter values in control plants were high even at low and medium oil concentrations. At the highest oil concentration (12%), parameter values of this cluster sharply decreased and were minimal.

The second cluster included such parameters as root length, root fresh weight, root dry weight, chlorophyll *a* content, chlorophyll *b* content, and total chlorophyll content. These indicators were the most sensitive to oil pollution. They were characterized by a sharp decrease even at relatively low oil concentrations (1.5–3.0%).

The third cluster included parameters such as ascorbic acid content, ascorbate peroxidase activity, peroxidase activity, and glutathione peroxidase activity. Parameter values of this cluster increased in comparison with the control at low or medium oil concentrations in the soil and decreased at high concentrations, respectively.

The fourth cluster included such parameters as malondialdehyde content, hydrogen peroxide content, proline content, 2,3-diketogulonic acid content, dehydroascorbic acid content, phenolic compounds content, and superoxide dismutase activity. Parameter values of this cluster were minimal in the control plants and increased with an increase in the oil concentration in the soil.

Distribution of plant samples into clusters primarily depended on the oil concentration in the soil rather than rye variety (Figure 6, at the left). The first cluster included control plants of both varieties and Valdai plants at 1.5% oil concentration in the soil. The second cluster included Krona plants at 1.5% and 3% oil concentrations. The third cluster included Valdai plants at 3% and 6% concentrations. The fourth cluster included Krona plants at 6% oil concentration and Valdai plants at 12% oil concentration. A separate cluster was formed by Krona plants grown at 12% oil concentration in the soil. These plants were characterized by minimal or very low parameter values of growth, chlorophylls content, carotenoids content, ascorbic acid content, non-protein thiols content, activities of CAT, APX, POD, and GPX, as well as high values of malondialdehyde content, hydrogen peroxide content, oxidized forms of ascorbic acid (dehydroascorbic and 2,3-diketogulonic acids) content, phenolic compounds content, and superoxide dismutase activity.

## 3. Discussion

### 3.1. Difference in Growth Response of Two Rye Varieties to Oil Pollution

The effect of crude oil on plants depends on a number of factors, among which the plant species and plant variety prove to be of great importance. The current study was devoted to investigating the effect of different oil concentrations on two rye varieties–Krona and Valdai. Previous studies have shown that these varieties differ in winter hardiness and their resistance to low temperatures, acidification, and high content of aluminum ions in the soil [31,32,33]. The current study revealed that a significant decrease in shoot biomass was observed in the Krona variety at 6% oil concentration in the soil (by 24% lower compared to the control) and in the Valdai variety at 12% oil concentration in the soil (by 25% lower compared to the control). The decrease in Krona shoot biomass was about 57% compared to the control at 12% oil concentration in the soil.

At the same time, a stimulating effect of low oil concentrations (1.5 and 3%) on the biomass growth of Valdai variety was detected. Similar results on the dual effect of oil on plant growth and development were obtained by Ayotamuno and Kogbara [27] while investigating the effects of different oil concentrations on maize plants. The authors revealed that maize could survive soil pollution of about 21% and produce fresh cob yield of about 60% than on the normal soil. At a lower level of soil contamination with oil (12.5%), there was a stimulated increase in the fresh cob yield of maize. This stimulating effect could be attributed to the bacterial breakdown of the hydrocarbons, release of nutrients from the oil, or hormonal influence [34]. However, further research is needed for more accurate identification of the reasons for the stimulating effect of oil on plants.

Root biomass turns out to be the main indicator of the plant’s ability to grow on the oil-polluted soil, since the biodegradation of hydrocarbon contaminants is particularly active in the rhizosphere. However, the current study revealed that with an increase in the oil concentration in the soil, root biomass and root length of both rye varieties sharply decreased, and appeared to be 3–4 times less than in the control at 12% oil concentration in the soil. A decrease in root biomass of plants growing on oil-polluted soils was also shown in [35,36,37].

The main factors of the negative effect of soil oil pollution on plants appear to be direct toxic effect of petroleum hydrocarbons and indirect effects associated with changes in physicochemical and biochemical properties of the soil, as well as with the destruction of soil microbial communities and reduction in the diversity and the quantity of soil organisms [38]. The indirect effect of oil soil pollution on the physiological state of plants can also be determined by the fact that oil triggers water-stress, osmotic stress, anaerobic stress, and nutrient deficiency stress in plants via soil–root–plant interaction [34]. This statement is also confirmed by the results of transcriptome analyses of *Z. mays* presented in [39]. The analyses show that the metabolism of water uptake and osmotic homeostasis-related gene expression is predominantly influenced by the petroleum hydrocarbon treatment. However, it should be noted that the current study revealed that water content in shoots and roots of both rye varieties proved to be significantly lower only at the maximum oil concentration in the soil (12%) and did not differ from the control at lower concentrations.

Thus, the study of growth-related attributes of two rye varieties revealed that Valdai compared to Krona variety was more resistant to oil pollution.

### 3.2. Changes in Biochemical Parameters of Rye under Crude Oil-Induced Stress

According to the results of the cluster analysis (Figure 6), the studied biochemical parameters were divided into four clusters depending on their response to increasing the concentration of crude oil in the soil.

One of the clusters included chlorophylls, the content of which sharply decreased in rye shoots of both varieties with an increase in the oil concentration in the soil. It is known that the photosynthetic apparatus of plants in general and their chlorophyll level in particular are very sensitive to unfavorable factors. Despite the fact that, in some studies, it was shown that content of chlorophylls is not an accurate tool for forecasting plant response to environmental stress [40], the content of photosynthetic pigments in leaves turns out to be an indirect indicator reflecting the efficiency of photosynthesis. As shown in previous research [41], mechanisms of inhibitory effects of polycyclic aromatic hydrocarbons on photosynthetic processes includes damage to the lipid bilayer of the plasma membrane and then violation of lipid bilayer membranes of cell organelles and generation of ROS. As a result, this leads to a violation in the PS-2 antennae complex, increase in the content of PS-2 QB-non-reducing complexes, enhancement of heat dissipation of absorbed energy in PS-2, and to a decrease in content of photosynthetic pigments [41].

The next cluster included the parameters of oxidative stress (MDA, H_2_O_2_), oxidized forms of ascorbic acid, some non-enzymatic antioxidants (proline and phenolic compounds) and SOD.

Intensification of free radical accumulation in cells and oxidative stress development occur when plants are exposed to various unfavorable environmental factors, such as drought, deficiency of mineral nutrients, high intensity of light, soil contamination with heavy metals [42]. The current study revealed that soil pollution with oil led to an increase in contents of H_2_O_2_ and MDA in rye shoots of both varieties. The results obtained are consistent with the previously published data on the effect of oil pollution on active production of reactive oxygen species and peroxidation in mangrove [43], cowpea and maize [7] plants.

Accumulation of dehydroascorbic and 2,3-diketogulonic acids at high oil concentrations can be also considered as an evidence of the shift in redox processes in rye plant cells towards oxidation. The oxidation of AsA to DHA is reversible; however, DHA can be irreversibly hydrolyzed to 2,3-diketogulonic acid. The content of 2,3-diketogulonic acids in plant tissues is an indicator of the process direction in the AsA-DHA system. It depends on the activity of enzymes that oxidize AsA (for example, ascorbate peroxidase and ascorbate oxidase), the activity of enzymes involved in DHA reduction (dehydroascorbate reductase and monodehydroascorbate reductase), and the presence of reducing agents such as glutathione and NADPH in the cell [44].

Regulation of redox-homeostasis in plants in the conditions of technogenic pollution is based on activation of the antioxidative system, which includes a number of low-molecular weight compounds and antioxidative enzymes [16,45]. Some authors suppose that the enzymatic system provides the most effective protection of plant metabolic processes from ROS while other authors think that this role is played by low-molecular weight antioxidants [46,47]. In the current study, the strongest positive correlation between contents of MDA and H_2_O_2_ and contents of proline and phenolic compounds as well as superoxide dismutase activity was detected (r = 0.81–0.95, *p* ≤ 0.05).

Proline accumulation in plants can regulate the osmotic potential of cells, stabilize the cell structure, and remove excess reactive oxygen species, thereby improving the resistance of rye to environmental stress [31,48]. The current study revealed that proline content in rye shoots increased with an increase in the oil concentration in the soil. Furthermore, these changes were more pronounced in the Krona variety. Despite the fact that the current experiment did not reveal any significant changes in water content in rye shoots grown on the oil-polluted soil, the increase in proline content in plants can be related to its functioning as an indicator of water stress. Data on changes in proline content in plants at different oil concentrations in the soil are rather controversial and are determined by both the pollutant type and plant species. For example, it was previously shown that soil contamination with heavy metals, as a rule, leads to an increase in proline content in plants [49,50]. Additionally, a number of studies revealed a decrease in proline level in leaves of *Plantago lanceolata* [51] and *Brassica juncea* [52] exposed to heavy metal soil contamination. Rusin et al. [17] found that soil contamination with petroleum-derived substances led to a decrease in proline content in leaves of broad bean plants. At the same time, the authors previously showed that soil contamination with diesel and gasoline led to an increase in proline content in wheat plants [5].

Similar to proline, content of phenolic compounds in both rye varieties increased with an increase in the oil concentration of soils. Plants are known to enhance the synthesis of polyphenols such as phenolic acids and flavonoids under abiotic stress conditions (drought, salinity, high/low temperature, ultraviolet radiation, and heavy metals) that enables them to resist to adverse environmental factors. The biosynthesis of phenolics under stressful environments is regulated by the altered activities of various key enzymes of phenolic biosynthetic pathways and by the up-regulation of the transcript levels of genes encoding these enzymes [53]. In conditions of soil oil pollution, the induction of phenolic compounds biosynthesis in plants can be determined by an increase in ROS content and by a deficiency of mineral nutrients such as nitrogen or phosphorus, which is observed at a high oil concentration in the soil [34]. To the best of our knowledge, there have been no previous studies on the change in the gene expression of phenylpropanoids biosynthesis in the conditions of crude oil-polluted soil. Further studies will make it possible to broaden the understanding of the mechanisms of the secondary plant metabolism response to the oil-polluted soil.

It is known that plant resistance to free radicals is also associated with an increase in the activities of antioxidative enzymes after exposure to pollutants [44,54]. The current study revealed an increase in SOD activity in shoots of both rye varieties with an increase in the oil concentration in the soil, which indicated that antioxidative defense mechanisms were activated in the plant. However, the increase in SOD activity was insufficient at high oil concentrations in the soil, since the peroxidation level remained high enough which was proved by the high content of malondialdehyde in these plants.

Two other clusters included non-enzymatic antioxidants (non-protein thiols, ascorbic acid) and the most of the studied antioxidative enzymes (CAT, APX, GPX, POD). The contents or activities of these antioxidants were maximal at low or medium concentrations of crude oil in the soil.

Non-protein thiols, such as glutathione and cysteine, are involved in several biological processes in the cell including the cell’s defense against the reactive oxygen species [55]. The current study determined a slight decrease in the content of non-protein thiols at low oil concentrations as well as stimulation of its accumulation at medium concentrations. At the maximum oil concentration in the soil, the content of non-protein thiols was minimal in shoots of both rye varieties. On the one hand, the result obtained can be attributed to activation of glutathione biosynthesis and cysteine biosynthesis in the plant qualitative response to moderate stress. On the other hand, at high oil concentrations, non-protein thiols are likely to be consumed much faster compared to its synthesis. Studies of the effect of different cadmium concentrations on *Eichhornia crassipes* [56] and arsenic concentrations on *Wrightia arborea* [57] demonstrated the similar results.

Together with glutathione, ascorbic acid is an important component of the Foyer–Halliwell–Assad cycle of plant antioxidative defense [58]. The revealed increase in the content of ascorbic acid in shoots of both rye varieties indicates that ascorbic acid plays an important role in cellular ROS homeostasis regulation during rye plants adaptation to crude oil-polluted soil. Sharper change in AsA in Valdai shoots compared to Krona is likely to determine the greater resistance of Valdai variety to moderately oil pollution of the soil.

The response of most of the studied enzymes (CAT, APX, and GPX) to soil contamination with crude oil depended on the oil concentration and rye variety. Thus, compared to the control, an increase in activities of these enzymes was revealed only at certain oil concentrations (for example, for catalase at 1.5–6.0% and for glutathione peroxidase at 1.5% in the Krona variety, and at 1.5 and 3% in the Valdai variety, respectively). Dependence of catalase activity changes on the oil concentration in the soil was previously shown in the study on jojoba plants [12]. Unlike all other studied enzymes, activity of peroxidase under soil oil contamination was lower or equal to that of the control. Due to the fact that antioxidative enzymes are only part of a complex finely regulated antioxidative system of plant defense against adverse environmental factors, the changes in their activity or gene expression, as a rule, depends on species and variety of plants, type of an impact, strength (dose) of an impact, and duration of exposure.

Under abiotic stress caused by pollutions the activity and gene expression of antioxidative enzymes or the key enzymes of biosynthetic pathways of non-enzymatic antioxidants can be regulated via signaling molecules such as phytohormones, reactive oxygen species (ROS) and nitric oxide [16]. According to the published papers, the data on activation or inactivation of a certain signaling pathway and molecules (mitogen-activated protein kinases (MAPKs), phosphatase, calcium (Ca^2+^)) in plants in conditions of oil-polluted soil have been insufficient so far. Conducting further studies will contribute to more detailed understanding of molecular mechanisms of the response and possible adaptation of plants to the crude oil-polluted soil.

## 4. Materials and Methods

### 4.1. Chemicals and Reagents

Folin–Ciocalteu reagent, 2,4-dinitrophenylhydrazine, 2-thiobarbituric acid, 5,5′-dithiobis- (2-nitrobenzoic) acid, bovine serum albumin (BSA), Coomassie Brilliant Blue G-250, gallic acid, guaiacol, L-ascorbic acid, L-glutathione reduced, L-methionine, L-proline, nitro blue tetrazolium, polyvinylpyrrolidone, riboflavin, trichloroacetic acid were purchased from Sigma (St. Louis, MO, USA). All other reagents and solvents were analytical grade from Vecton (Saint Petersburg, Russia).

### 4.2. Plant Materials and Growth Conditions

The pot experiment was conducted in the glass covered greenhouse at Immanuel Kant Baltic Federal University (Kaliningrad, Russia, 54°44′ N 20°30′ E), from 16 April to 28 May 2018. The Krona and Valdai varieties of winter rye (*Secale cereale* L.) used in the experiment were obtained from the Seed station at the Ministry of Agriculture of the Kaliningrad region. These varieties were chosen for two reasons. Firstly, both varieties are listed in the *State Register for Cultivation in the North-Western Region of Russia* including the Kaliningrad Region located in the Southeastern Baltic coast. The climate of Region is temperate, marine transitional to continental (the July mean temperature ranges from +17 to +18 °C; the January mean temperature is −4 to −2°C; the average annual precipitation is 750 mm). Secondly, it was previously shown that these varieties differ in their resistance to particular abiotic factors (low temperatures, acidification, and high concentration of aluminum ions in the soil). Rye was grown in the culture pots filled with the soil collected from the field located in Bagrationovskiy District (the Kaliningrad Region, Russia, 54°29′ N 20°25′ E). The soil used in the experiment had the following physicochemical properties: soil texture–clay clam, pH-6.7, organic matter–1.76%, total N content–0.23%, available P–17.8 mg kg^−1^, available K–212 mg kg^−1^. The pot dimensions were 29 cm height and 20 cm radius. Each pot contained 10 kg of soil. Crude oil was introduced into the soil to yield the concentrations of 1.5, 3.0, 6.0, 12.0% (i.e., 15, 30, 60 and 120 g crude oil per 1 kg soil). Crude oil effect on the plants was compared to the control (without oil introduction). Crude oil used in the experiment came from the oilfield located in the Kaliningrad region. Its API (American Petroleum Institute) gravity was 35.2, and its sulfur content was equal to 0.2% (*w/w*). Oil used in the experiment consisted of 1.05% asphaltene, 66.7% aliphatic hydrocarbons, 21.7% aromatic hydrocarbons, and 10.54% polar materials. Moreover, the following basic fertilizers were added: 0.17 g of nitrogen in terms of ammonium nitrate, 0.13 g of P_2_O_5_ in terms of potassium hydrophosphate, 0.28 g of K_2_O in terms of potassium hydrophosphate and sulfate, and 0.044 of MgO in terms of magnesium sulfate per kg of the soil. Solutions of fertilizers and crude oil were introduced in such a manner that they could not mix with each other. At the start of the experiment, basic elements (phosphorus, potassium, nitrogen, and magnesium) were introduced into the extreme points of the square on the soil surface, whereas crude oil was applied to its central point. After air-drying, the soil was mixed, and rye was planted. There were 20 plants in each pot. During the experiments, each pot was weighed and the plants were watered every 3 days using deionized water to maintain the soil moisture at approximately 70% of the soil’s water-holding capacity. Average air temperature in the greenhouse during the growing period was 18.4 °C and relative humidity was on average 68% (SHT71 temperature and humidity sensor, Sensirion AG, Stäfa, Switzerland), photosynthetic photon flux density was of 350 µmol m^−2^ s^−1^ (Li-250A lightmeter, Li-COR, Lincoln, NE, USA) at the plant height. Four replications for each treatment (crude oil introduction and rye varieties) were conducted. There were 40 pots in total. The pots were arranged in a completely randomized design.

### 4.3. Plant Harvesting and Sample Preparation

Plant harvesting for analysis was performed at tillering growth stage (GS 26, main shoot and 6 tillers) according to Zadoks’ scale. After harvesting, shoot fresh weight and root fresh weight from each pot were measured. The plant height and root length were measured for ten randomly selected plants from each pot. One part of the shoots was dried and used to determine water content and shoot dry weight. The whole root biomass was dried at 60 °C for 48 h. Then, root dry weight was measured. For biochemical analysis, fresh shoots were placed in liquid nitrogen within 5–10 min after harvesting and stored at −80 °C.

### 4.4. Analysis of the Biochemical Parameters of the Plants

#### 4.4.1. Photosynthetic Pigments

Accurately weighted 0.5 g plant sample was taken and homogenized in tissue homogenizer (Ultra-Turrax Tube Drive, IKA, Staufen, Germany) with 10 mL of 80% acetone. The homogenized sample mixture was centrifuged at 10,000 rpm for 15 min at 4 °C. The supernatant was separated. Then, 0.5 mL of it was mixed with 4.5 mL of the solvent. Optical absorbance of the above mixture was determined at 470 nm, 646.8 nm, and 663.2 nm. Pigments content was calculated according to [59]. Finally, contents of chlorophyll *a*, chlorophyll *b*, and carotenoids in plant were converted to mg per gram dry weight. The total chlorophyll content was calculated by adding contents of chlorophyll *a* and chlorophyll *b*.

#### 4.4.2. Malondialdehyde

Homogenization of the plant material (0.5 g) was performed using a cooled mortar and pestle in a mixture of 20% polyvinylpyrrolidone and 0.1% trichloroacetic acid. The homogenate was centrifuged at 10,000× *g* for 10 min under 4 °C. The malondialdehyde concentration in the supernatant was determined by reaction with thiobarbituric acid as described in [60]. Briefly, the supernatant was added to 5% thiobarbituric acid solution in 20% trichloroacetic acid solution and incubated in a water bath at 95 °C for 30 min. Then, the reaction was stopped by placing the tubes on ice for 10 min. The mixture was centrifuged at 10,000× *g* for 10 min. Optical absorbance of the supernatant was determined at 532 and 600 nm. Malondialdehyde content in the plant material was calculated using an extinction coefficient of 155 mM^−1^ cm^−1^ and expressed in nmol per gram dry weight.

#### 4.4.3. Hydrogen Peroxide

Hydrogen peroxide content in rye shoots was determined according to [61]. The plant material was homogenized in trichloroacetic acid in an ice bath. The homogenate was centrifuged at 16,000× *g* for 15 min. The supernatant was added to the reaction mixture containing 10 mM of phosphate buffer (pH 7.0) and 1 M of potassium iodine (KI). The mixture was incubated in the dark for 1 h. Optical absorbance was measured at 390 nm. Hydrogen peroxide content in the plant material was determined using a calibration graph and expressed in nmol H_2_O_2_ per gram dry weight.

#### 4.4.4. Proline

Proline content in rye shoots was determined spectrophotometrically using the acid-ninhydrin method as described in [62] with some modifications. The plant material (0.5 g) was homogenized in 10 mL of 3% sulfosalicylic acid. The homogenate was filtered, and the filtrate was used for further analysis. The filtrate was mixed with 2 mL of ninhydrin reagent (1.25 g of ninhydrin, 30 mL of glacial acetic acid, 20 mL of 6 M H_3_PO_4_ solution) and 2 mL of glacial acetic acid. The reaction mixture was incubated for 1 h in a water bath at 100 °C, after which it was rapidly cooled on ice. The mixture was extracted with toluene, and optical absorbance was determined at 520 nm. Standard solutions of L-proline were used to make the calibration graph. Proline content in the plant material was expressed in µmol per gram dry weight.

#### 4.4.5. Non-Protein Thiols

Non-protein thiols content was determined using 5,5′-dithiobis- (2-nitrobenzoic acid) as described in [63]. The plant material (1 g) was homogenized in 10 mL of 5% sulfosalicylic acid. The homogenate was centrifuged at 20,000× *g* for 20 min. The reaction mixture contained supernatant, 0.1 M sodium phosphate buffer (pH 7.0), 0.5 mM EDTA, and 0.25 mM 5,5′-dithiobis- (2-nitrobenzoic acid). The mixture was incubated for 10 min at room temperature. Optical absorbance was measured at 412 nm. Non-protein thiols content was determined using a calibration graph. Glutathione was used as a standard. Non-protein thiols content in plants was expressed in μmoles per g dry weight.

#### 4.4.6. Ascorbic, Dehydroascorbic and 2,3-Diketogulonic Acids

Contents of ascorbic acid, dehydroascorbic acid, and 2,3-diketogulonic acid were measured spectrophotometrically by using the reaction of DHA and DKGA with 2,4-dinitrophenylhydrazine as described in [44]. To find the total content of all acids, AsA was oxidized with 2,6-dichlorophenolindophenol reagent (2% solution in 4.5 M sulphuric acid, containing 0.25% of thiourea). In order to determine DHA and DKGA separately, DHA in the plant extract was reduced to AsA with 2·10^−3^ M unithiol solution prepared in phosphate buffer (pH 7.0). This step made it possible to determine DKGA content. DHA content was calculated as difference between results obtained without the “reduction” step and with it. Finally, ascorbic acid content was calculated as difference between the total contents of the three acids, and the sum of contents of DHA and DKGA. Ascorbic acid solutions of fixed concentration were used to make a calibration curve. Contents of AsA, DHA and DKGA were expressed in μg of per gram dry weight.

#### 4.4.7. Total Phenolic Compounds

Total phenolics content was determined using Folin–Ciocalteu method as described in [44]. Briefly, the plant material (0.1 g) was homogenized in 10 mL of 70% ethanol. The homogenate was centrifuged at 4500× *g* for 30 min. The reaction mixture contained 100 µL of supernatant, 300 µL of Folin–Ciocalteu reagent, and 6 mL of 6.75% solution of sodium carbonate. The mixture was incubated for 30 min in the dark at room temperature. Optical absorbance was measured at 720 nm. Content of total phenolic compounds was determined using a calibration curve with gallic acid as standard and expressed in mg gallic acid equivalents per g dry weight.

#### 4.4.8. Antioxidative Enzymes Activity

Samples of frozen shoots (approximately 0.4 g of fresh weight) were ground in liquid nitrogen and homogenized in 2.0 mL of ice-cold 100 mM phosphate buffer containing 0.1 mM EDTA and 1.0% polyvinylpyrrolidone. The homogenate was centrifuged at 12,000× *g* for 20 min at 4 °C. The supernatant was used to analyze the activities of antioxidative enzymes and protein content.

Superoxide dismutase (SOD, EC 1.15.1.1) activity was determined by its ability to inhibit the photochemical reduction of nitro blue tetrazolium (NBT) as described in [64] with some modifications. The reaction mixture contained 63 µM NBT, 13 mM L-methionine, 0.1 mM EDTA, 0.05 M sodium carbonate and 0.5 mL enzyme extract (or distilled water in control). The reaction was started by adding 20 μL of 0.025% riboflavin. The tubes were then quickly placed under fluorescent lamps (18 W). The reaction time was 15 min. After the time elapsed, the reaction was stopped by placing the tubes in the dark. Optical absorbance of solutions was determined at 560 nm. The amount of enzyme necessary to inhibit the photoreduction of 50% nitro blue tetrazolium at 25 °C was taken as a unit of SOD activity and was expressed per mg of protein.

Catalase (CAT, EC 1.11.1.6) activity was determined by the decrease in optical absorbance at 240 nm caused by H_2_O_2_ decomposition [65]. The reaction mixture contained 2.95 mL of 50 mM K, Na-phosphate buffer (pH 7.0), and 30 μL of the plant extract. The reaction was started by introducing 20 μL of 0.6 M hydrogen peroxide into the reaction mixture. The control cuvette contained the same reagents, but no hydrogen peroxide was added. Catalase activity was determined by the change in optical absorbance at 240 nm every second for 100 s. Extinction coefficient (ε = 39.4 mM^–1^ cm^–1^) was used for calculations.

Ascorbate peroxidase (APX, EC 1.11.1.11) activity was determined by monitoring the oxidation rate of H_2_O_2_-dependent ascorbate as described in [66]. The reaction mixture included 80 mM phosphate buffer (pH 7.0), 0.6 mM H_2_O_2_, 0.1 mM Na_2_EDTA, 0.5 mM ascorbic acid and 50 μL of the plant extract. H_2_O_2_ was added to start the reaction at 25 °C. The decrease in optical absorbance of the mixture was measured at 290 nm every second for 120 s. Enzyme activity was calculated based on extinction coefficient equal to 2.8 mM^−1^ cm^−1^.

Glutathione peroxidase (GPX, EC 1.11.1.9) activity was determined according to [67] using hydrogen peroxide as a substrate. To perform the enzymatic reaction, 200 μL of the plant extract was placed into the reaction tube containing 400 μL of 0.1 mM solution of reduced glutathione and 200 μL of 0.067 M solution of KNaHPO_4_. The mixture containing all the reagents except the plant extract was used as a control. After incubating the mixture in a water bath at 25 °C for 5 min, 0.2 mL of 1.3 mM hydrogen peroxide solution was added to the mixture. The reaction time was 10 min. The reaction was stopped by adding 1 mL of 1% trichloroacetic acid solution and placing the reaction mixture in an ice bath. Then, the mixture was centrifuged and used to determine glutathione content in it. For determining glutathione content, 0.48 mL of the supernatant was mixed with 2.2 mL of 0.32 M Na_2_HPO_4_ and 0.32 mL of 1.0 mM 5,5′-dithiobis-(2-nitrobenzoic) acid. The mixture was incubated for 10 min at room temperature. Optical absorbance was measured at 412 nm. Enzyme activity was calculated based on the decrease in content of reduced glutathione.

Peroxidase (POD, EC 1.11.1.7) activity was determined spectrophotometrically using guaiacol as a phenolic substrate and hydrogen peroxide [68]. The reaction mixture contained 0.15 mL of 4% guaiacol, 0.15 mL of 1% (*v/v*) H_2_O_2_, 2.66 mL of 0.1 M phosphate buffer (pH 7.0), and 40 μL of the plant extract. The control sample contained all the reagents but the plant extract. Optical absorbance of the mixture was measured at 470 nm. Enzyme activity was calculated based on extinction coefficient of tetraguaiacol equal to 26.6 mM^–1^ cm^–1^.

Activities of all studied antioxidative enzymes were converted to mg of protein. Total soluble protein was estimated according to the Bradford method [69] with bovine serum albumin (BSA) as a standard. Shimadzu UV-3600 spectrophotometer (Shimadzu, Kyoto, Japan) was used for spectrophotometric analyses.

### 4.5. Statistical Analysis

Experimental data were statistically processed using the SigmaPlot 12.3 (Systat Software GmbH, Erkrath, Germany), OriginPro 9 (OriginLab Corporation, Northampton, MA, USA) and RStudio Software. The tables and graphs show the mean values with the standard deviation (*n* = 4). To estimate statistically significant differences between the experimental variants, the two-way analysis of variance (two-way ANOVA) was performed. A one-way analysis of variance (one-way ANOVA) was performed for each factor (the oil concentration and the rye variety) separately because the two-factor analysis revealed a significantly influential interaction between the factors (Table 1, Appendix A, Table A1 and Table A2). Tukey’s test was used as a criterion of the significance of differences at *p* ≤ 0.05 significance level. Pearson correlation was applied to analyze the correlation between the studied parameters and correlation plot was constructed with reference of coefficient correlation values. The heat map and clusters were constructed based on normalized values of studied parameters. The Euclidean distance was used as a similarity measure.

## 5. Conclusions

Based on the results of a comprehensive analysis of growth and biochemical parameters and of cluster analysis, the Valdai variety proved to be more resistant to oil pollution. In general, a weaker decrease in growth parameters and in contents of chlorophyll *a* and total chlorophylls, as well as an increase in contents of lipid peroxidation products and oxidized forms of ascorbic acid were observed in the Valdai compared to the Krona variety at higher soil oil concentrations. At the same time, an increase in the contents of some low-molecular antioxidants (proline, non-protein thiols, ascorbic acid, and phenolic compounds) and an increase in activities of the antioxidative enzymes (SOD, CAT, APX, and GPX) were observed at different oil concentrations in the soil in both rye varieties. In order to determine the specific response of plants to oil pollution, it is necessary to conduct further studies including the study of petroleum hydrocarbons accumulation in plants, changes in the membrane structure, expression of stress-related genes and genes involved in the plant secondary metabolism, changes in phytohormones functioning and signaling pathways.

The results of the current study reveal that Valdai variety plants are able to grow on moderately oil-polluted soils (up to 3% oil concentration) without loss in growth and weight. Based on this, Valdai variety is considered to be a promising rye variety for cultivation on moderately oil-polluted soils with the aim of their decontamination. At the same time, further studies should be undertaken in order to investigate the processes of oil transformation in the soil-rye system with the consideration of the soil and rhizosphere microbiomes.

## Figures and Tables

**Table 1:** Effect of different concentrations of crude oil in the soil on growth-related attributes of two rye varieties.

Variety	Oil Concentration	Plant Height, cm	Shoot Fresh Weight, g Plant^−1^	Shoot Dry Weight, g Plant^−1^	Water Content in Shoots, %	Root Length, cm	Root Fresh Weight, g Plant^−1^	Root Dry Weight, g Plant^−1^	Water Content in Roots, %
Krona	0	17.20a ^1^	0.738a	0.073a	90.1a	8.22a	0.338a	0.026a	90.1a
	1.5	16.91a	0.770a	0.074a	90.4a	6.62b	0.283b	0.022b	90.4a
	3.0	16.82a	0.708a	0.068b	90.4a	4.59c	0.174c	0.018c	90.4a
	6.0	14.08b	0.564b	0.062c	88.9a	3.58d	0.133d	0.016c	88.9a
	12.0	9.58c	0.319c	0.044d	86.1b	2.35e	0.082e	0.012d	86.1b
Valdai	0	18.17b	0.797ab	0.073b	90.8a	8.38a	0.379a *	0.031a *	90.8a
	1.5	19.93a *	0.857a *	0.087a *	89.9a	7.62b *	0.349b *	0.028a *	89.9a
	3.0	19.82a *	0.874a *	0.084a *	90.4a	6.03c *	0.270c *	0.023b *	90.2a
	6.0	18.02b *	0.715b *	0.072b *	89.9a	4.50d *	0.170d *	0.018c *	89.9a
	12.0	14.95c *	0.597c *	0.077b *	87.1b	3.18e *	0.114e *	0.014d	87.1b
Main effects ^2^									
Oil concentration	0	17.70bc	0.767a	0.0731b	90.4a	8.30a	0.358a	0.0285a	92.0a
(O)	1.5	18.41a	0.814a	0.0801a	90.1a	7.12b	0.316b	0.0252b	92.1a
	3.0	18.30ab	0.791a	0.0759b	90.3a	5.31c	0.222c	0.0203c	90.7b
	6.0	16.04c	0.639b	0.0671c	89.4a	4.04d	0.151d	0.0169d	88.8c
	12.0	12.26d	0.458c	0.0605d	86.6b	2.77e	0.098e	0.0125e	87.0d
Rye variety	Krona	14.91b	0.620b	0.0641b	89.2a	5.07b	0.202b	0.0186b	89.7b
(V)	Valdai	18.17a	0.768a	0.0786a	89.6a	5.94a	0.256a	0.0227a	90.5a
Significance	O	*	*	*	*	*	*	*	*
	V	*	*	*	ns	*	*	*	ns
	O*V	*	*	*	ns	*	*	ns	*

^1^ Different lower-case letters indicate significant differences between plants by oil concentration in the soil for each variety separately, and asterisks * indicate significant differences between rye varieties at *p* ≤ 0.05 based on post hoc Tukey’s test (*n* = 4) ^2^ Data were evaluated via two-way ANOVA, factors: oil concentration (%) and rye variety, followed by Tukey’s HSD test (*n* = 4, *p* ≤ 0.05). Identical letters indicate that values do not differ significantly. Asterisks indicate significantly influential factors. ns–not significantly influential factors.

## Data Availability

The data presented in this study are available on request from the corresponding author.

## Appendix A

**Table A1:** Results of two-way ANOVA for photosynthetic pigments and oxidative stress parameters.

Main Effects ^1^	Factors	Chl *a*	Chl *b*	Total Chl	Car	MDA	H_2_O_2_
Oil concentration	0	0.658a	0.281a	0.939a	0.192a	105.9d	516.5c
(O)	1.5	0.626b	0.268b	0.894b	0.192a	108.3d	522.1c
	3.0	0.585c	0.255c	0.840c	0.197a	117.3c	573.6b
	6.0	0.548d	0.254c	0.802d	0.195a	144.5b	589.3b
	12.0	0.535d	0.255c	0.790d	0.171b	173.0a	724.3a
Rye variety	Krona	0.572b	0.261a	0.834b	0.178b	138.1a	629.6a
(V)	Valdai	0.609a	0.264a	0.872a	0.201a	121.5b	540.8b
Significance	O	*	*	*	*	*	*
	V	*	ns	*	*	*	*
	O*V	*	*	*	ns	*	*

^1^ Data were evaluated via two-way ANOVA, factors: oil concentration (%) and rye variety, followed by Tukey’s HSD test (n = 4, *p* ≤ 0.05). Identical letters indicate that values do not differ significantly. Asterisks indicate significantly influential factors. ns—not significantly influential factors. Chl *a*—chlorophyll *a*, Chl *b*—chlorophyll *b*, Total Chl—total chlorophylls, Car—carotenoids, MDA—malondialdehyde.

**Table A2:** Results of two-way ANOVA for non-enzymatic and enzymatic antioxidants.

Main Effects ^1^	Factors	Proline	-SH	AsA	DHA	DKGA	TPC	SOD	CAT	APX	GPX	POD
Oil concentration	0	15.38c	50.89b	159d	49.8d	59.0e	2.95e	7.30a	111.6b	17.8d	1.17b	43.4a
(O)	1.5	15.81c	44.66c	231b	52.8d	67.1d	3.71d	10.08b	138.1a	21.9bc	1.79a	32.5c
	3.0	16.85c	51.84b	268a	62.0c	73.3c	4.54c	10.68c	141.7a	24.1ab	1.26b	41.2ab
	6.0	22.29b	56.89a	244b	82.6a	84.9b	5.64b	12.25d	139.8a	25.7a	1.13b	40.1b
	12.0	28.70a	35.94d	191c	70.1b	91.8a	6.26a	13.76e	96.7c	20.9c	0.94c	29.6c
Rye variety	Krona	20.49a	41.76b	180a	66.7a	80.7a	4.37b	11.52a	108.1b	18.2b	1.24a	29.5b
(V)	Valdai	19.12b	54.33a	257b	60.3b	69.8b	4.87a	10.11b	143.1a	26.0a	1.28a	45.2a
Significance	O	*	*	*	*	*	*	*	*	*	*	*
	V	*	*	*	*	*	*	*	*	*	ns	*
	O*V	*	*	*	*	*	*	*	*	*	*	*

^1^ Data was evaluated via two-way ANOVA, factors: oil concentration (%) and rye variety, followed by Tukey’s HSD test (n = 4, *p* ≤ 0.05). Identical letters indicate that values do not differ significantly. Asterisks indicate significantly influential factors. ns—not significantly influential factors. –SH—non-protein thiols, AsA—ascorbic acid, DHA—dehydroascorbic acid, DKGA—2,3-diketogulonic acid, TPC—total phenolic compounds, SOD—superoxide dismutase, CAT—catalase, APX—ascorbate peroxidase, GPX—glutathione peroxidase, POD—peroxidase.
